# Microbial detoxification of lignocellulosic biomass hydrolysates: Biochemical and molecular aspects, challenges, exploits and future perspectives

**DOI:** 10.3389/fbioe.2022.1061667

**Published:** 2022-11-22

**Authors:** Victor C. Ujor, Christopher C. Okonkwo

**Affiliations:** ^1^ Metabolic Engineering and Fermentation Science Group, Department of Food Science, University of Wisconsin-Madison, Madison, WI, United States; ^2^ Biotechnology Program, College of Science, The Roux Institute, Northeastern University, Portland, ME, United States

**Keywords:** lignocellulosic biomass, lignocellulose-derived inhibitors, furfural, 5-hydroxymethylfurfural, solventogenic clostridium species, *Saccharomyces cereivisiae*, *Escherichia coli*

## Abstract

Valorization of lignocellulosic biomass (LB) has the potential to secure sustainable energy production without impacting food insecurity, whist relieving over reliance on finite fossil fuels. Agro-derived lignocellulosic residues such as wheat straw, switchgrass, rice bran, and miscanthus have gained relevance as feedstocks for the production of biofuels and chemicals. However, the microorganisms employed in fermentative conversion of carbohydrates to fuels and chemicals are unable to efficiently utilize the sugars derived from LB due to co-production of lignocellulose-derived microbial inhibitory compounds (LDMICs) during LB pretreatment. LDMICs impact microbial growth by inhibition of specific enzymes, cause DNA and cell membrane damage, and elicit cellular redox imbalance. Over the past decade, success has been achieved with the removal of LDMICs prior to fermentation. However, LDMICs removal by chemical processes is often accompanied by sugar losses, which negatively impacts the overall production cost. Hence, *in situ* removal of LDMICs by fermentative organisms during the fermentation process has garnered considerable attention as the “go-to” approach for economical LDMICs detoxification and bio-chemicals production. *In situ* removal of LDMICs has been pursued by either engineering more robust biocatalysts or isolating novel microbial strains with the inherent capacity to mineralize or detoxify LDMICs to less toxic compounds. While some success has been made along this line, efficient detoxification and robust production of target bio-chemicals in lignocellulosic hydrolysates (LHs) under largely anaerobic fermentative conditions remains a lingering challenge. Consequently, LB remains an underutilized substrate for bio-chemicals production. In this review, the impact of microbial LH detoxification on overall target molecule production is discussed. Further, the biochemical pathways and mechanisms employed for *in situ* microbial detoxification of furanic LDMICs [e.g., furfural and 5-hydroxymethylfurfural (HMF)] and phenolic LDMICs (e.g., syringaldehyde, *p*-coumaric acid, 4-hydroxybenzaldehyde, vanillin, and ferulic acid) are discussed. More importantly, metabolic engineering strategies for the development of LDMIC-tolerant and bio-chemicals overproducing strains and processes are highlighted.

## Introduction

**FIGURE 1:**
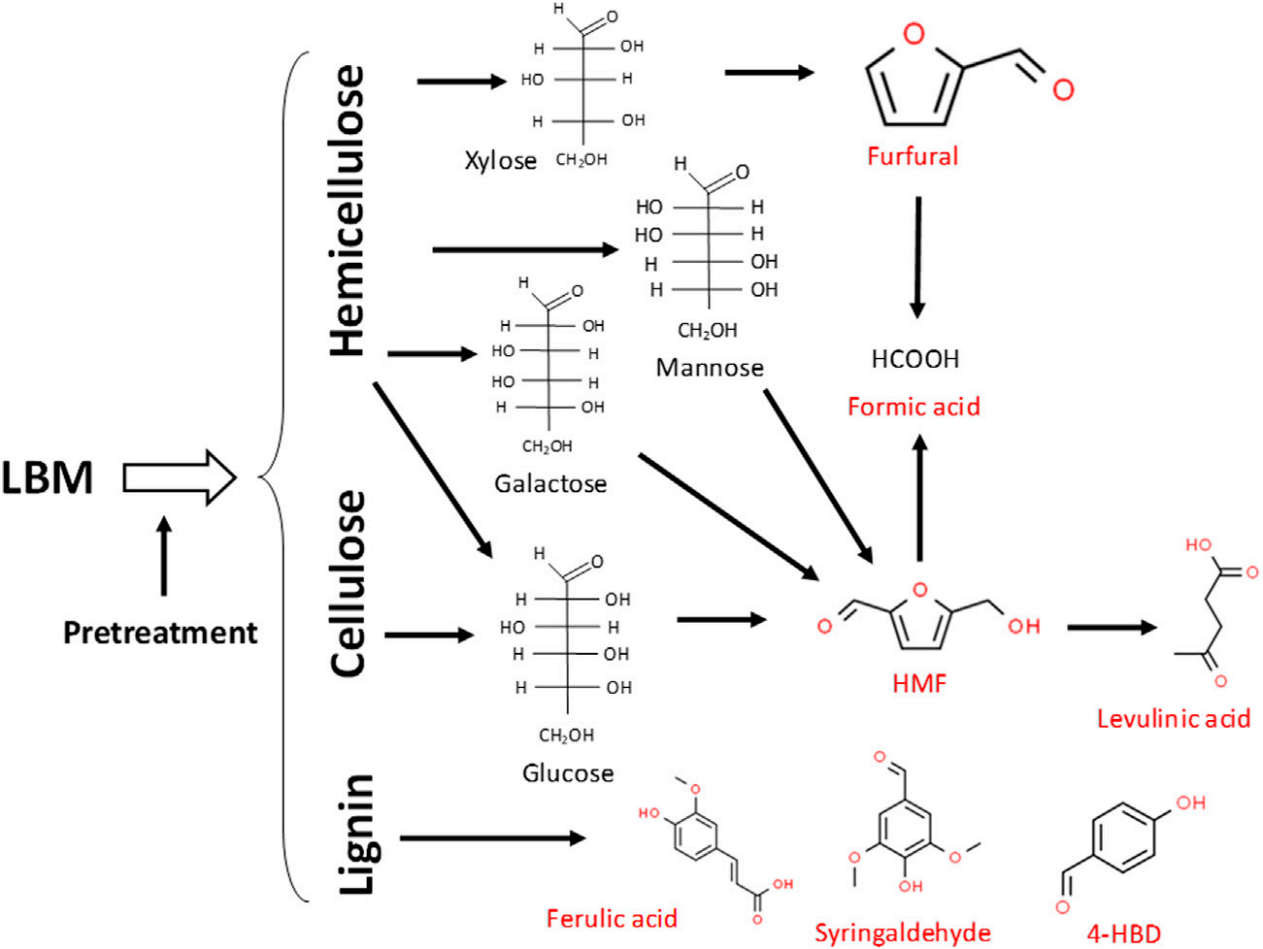
Major LDMICs that result from hemicellulose and cellulose and select lignin-derived inhibitors generated during dilute acid or hydrothermal pretreatment of LBMs. LDMICs are depicted in red. Inhibitor structures were obtained from the ChemSpider database (Available at: http://www.chemspider.com/).

Lignocellulosic biomass materials (LBMs) such wheat straw, corn stover, corn cobs, poplar, forest residues, rice bran and *Miscanthus giganteus*, among several other examples have enormous potential to supplant corn and other food-based feedstocks as the major sources of carbohydrates for fermentative production of fuels and chemicals (Palmqvist et al., 1999; Almeida et al., 2008; Almeida et al, 2009; Miller et al., 2009a; Miller et al., 2009b; Ujor et al., 2014a). However, despite extensive efforts, both at laboratory and industrial scales, cellulosic fuels and chemicals have yet to sufficiently take off. With increasing effects of climate change, it has become even more pressing to harness the sugars locked in LBMs as cheaper feedstocks for sustainable production of renewable chemicals. A major factor that impedes largescale deployment of LBMs in an industrial setting is co-generation of furanic and phenolic aldehydes and acids along with sugars, during pretreatment of LBMs (Figure 1; Palmqvist et al., 1999; Almeida et al., 2008; Almeida et al, 2009; Miller et al., 2009a; Miller et al., 2009b; Ujor et al., 2014b). Consequently, these aldehydes, phenolic compounds and acids, both separately and collectively, exert considerable toxicities on fermenting microorganisms, typically leading to premature termination of fermentation (Palmqvist et al., 1999; Sávári et al., 2003; Ujor et al., 2014a; Okonkwo et al., 2019, Okonkwo et al, 2021). Ultimately, this limits target product biosynthesis, which diminishes the economic competitiveness of LMBs-derived fuels and chemicals, relative to their oil-derived counterparts. Although chemical methods exist for the removal of furanic and phenolic lignocellulose-derived microbial inhibitory compounds (LDMICs) from lignocellulosic hydrolysates (LHs), they are often more expensive, and some are plagued by the loss of sugars during removal of the LDMICs (Nilvebrant et al., 2003; Alriksson et al., 2006; Alriksson et al., 2011; Zhang et al., 2011). This makes chemical remediation of LDMICs pre-fermentation economically unfavorable for largescale production of fuels and chemicals. Conversely, bioabatment, wherein, microorganisms—largely fermentative organisms—are used for *in situ* removal of LDMICs and concomitant production of the target products is deemed a more economical approach (Palmqvist et al., 1999; Almeida et al., 2008; Alriksson et al., 2011; Ujor et al., 2014b; Liu et al., 2015a; Agu et al., 2016; Okonkwo et al., 2019, 2021). Some success has been achieved *via* bioabatment in the forms of isolation or metabolic engineering of novel strains/species with enhanced capacities to detoxify lignocellulosic hydrolysates (LHs), or identification of metabolic perturbations that expedite the detoxification of LDMICs (Koopman et al., 2010; Wierckx et al., 2010; Ujor et al, 2015; Ujor et al., 2014a; Agu et al., 2016; Agu et al., 2018; Agu et al., 2019; Okonkwo et al., 2019; Okonkwo et al, 2021; Divate et al., 2022). Nonetheless, much needed additional studies are afoot towards identification or construction of superior strains with robust metabolic machineries for efficient detoxification/tolerance to LDMICs and target product accumulation. Additionally, there is need to explore a two-stage detoxification or a one-stage co-culture approach, whereby, one strain/species is deployed for the removal of LDMICs, followed or accompanied by fermentation with a dedicated target product-accumulating strain(s).

Different authors have reviewed the generation and removal of LDMICs, particularly furfural from LH. For instance, Palmqvist and Hahn-Hägerdal (2000) reviewed the LDMICs present in LH, the inhibitory mechanisms of LDMICs, and strategies for fermenting LHs with emphasis on yeast and ethanol production. Jönsson et al. (2013) reviewed the different LDMICs obtained during LBMs pretreatment and measures to alleviate inhibitor generation and toxicity to *Saccharomyces cerevisiae*. Chandel et al. (2013) discussed different mechanisms and methods for detoxification of LHs, including metabolic engineering approaches, albeit with emphasis on *S. cerevisiae* and ethanol production. A review by Ko et al. (2015) discussed the impacts of LDMICs on enzymes. Further, Jönsson and Martin. (2016) briefly examined the origins and chemical characteristics of LDMICs, with emphasis on their effects on microbial cells (largely on *Saccharomyces cerevisiae* and *Escherichia coli*) and cellulolytic enzymes, and measures for remedying their toxicities (including chemical detoxification). The central aim of this review is to broadly examine metabolic engineering approaches for remedying the toxicities of LDMICs in LHs, including *S. cerevisiae*, *E. coli*, and solventogenic *Clostridium* species—that have been largely under-reported in previous reviews—among other organisms. Furthermore, the roles of less or non-traditional fermenters and degraders of LDMICs in relation to LH conversion to different target chemicals, and the use of metabolic perturbations to accelerate inhibitor detoxification are discussed. We also highlight current limitations to bioabatement of LDMICs, whilst proffering likely future measures for improved removal of LDMICs, thus, enhanced fermentation of LHs into target products.

## Metabolic engineering and adaptive evolution strategies for enhanced fermentation of LHs to fuels and chemicals

### Saccharomyces cerevisiae

Towards detoxification of LDMICs, Larsson et al. (2001) engineered a strain of *S. cerevisiae* expressing a laccase gene from the white rot fungus *Trametes versicolor*. Laccases are largely fungal-derived extracellular blue multicopper oxidases that catalyze the oxidation of a wide assortment of phenolic, aromatic, and aliphatic amine substrates, leading to the reduction of molecular oxygen to water (Bassanini et al., 2021). When compared to the control strain not expressing a laccase gene, the laccase-expressing strain of *S. cerevisiae* achieved enhanced detoxification of spruce hydrolysate, leading to improved fermentation and ethanol production (Larsson et al., 2001). However, despite enhanced growth and fermentation, when compared to cultures grown in the absence of LDMICs (i.e., on glucose only), the laccase-expressing strain still exhibited a significant growth inhibition in the spruce hydrolysate. According to the authors, this effect likely stems from residual furanic aldehydes in the spruce hydrolysate (Table 1). Notably, whereas laccases are efficient at degrading phenolic compounds (Ujor et al., 2012; Asadgol et al., 2014), they are not as potent against furanic aldehydes such as furfural and 5-hydroxymethyl furfural (HMF), which together, account for most of the toxicities of LHs. Further, the laccase-expressing strain required adjustments of the culture pH—a key prerequisite of enzyme-catalyzed reactions—for improved fermentation of spruce hydrolysate (Larsson et al., 2001). At large scale, the need for pH adjustment will attract additional costs, which would derail the competitiveness of the resulting product. Therefore, there is need for additional investigations if this approach is to achieve both technical and economic success. First, deploying protein engineering to construct a laccase variant that is functional at the prevailing pH range during fermentation by *S. cerevisiae* will eliminate, or at least, significantly reduce the need for and ultimately, the cost associated with pH adjustment. Second, combined targeting of phenolic and furanic LDMICs—by engineering a robust furan-detoxifying or catabolizing metabolism alongside laccase-mediated removal of toxic phenols—may prove vastly successful towards largescale utilization of LBMs in biomanufacturing. Alternatively, a wider range of fungal species may be screened for laccases that are active at prevailing culture pH during fermentation by *S. cerevisiae*.

**TABLE 1:** Properties of select LDMICs and the mechanisms of microbial tolerance and detoxification.

Inhibitor class	LDMIC	Properties (*M*_*for*_; *Mr*; Log *Poctanol-water*)*^a^*	Mechanism of LDMIC tolerance/detoxification		Advantages	Disadvantages	References
Furanic aldehydes	Furfural	C_5_H_4_O_2_; 96.08; 0.73	Catalytic reduction	Reduction to their less toxic alcohols. Enzymes involved: short-chain dehydrogenase/red uctases (SDR), aldo-keto reductases (AKR), aldehyde reductase/dehydrog enase, alcohol dehydrogenases, and oxidoreductases	Eliminates/minimizes toxicity. Some microorganisms, (e.g., *C. basilensis*, *Bacillus* species) can further mineralize the alcohols	Accumulation of high concentrations of corresponding alcohols present a degree toxicity to microorganisms. Depletes NAD(P)H and ATP required for growth and product formation	^Palmqvist et al. (1999),^ ^Koopman et al. (2010),^ ^Ujor et al. (2014a),^ ^Zhang et al. (2015),^ ^Okonkwo et al. (2019)^
	HMF	C_6_H_6_O_3_; 126.11; −0.45	Catalytic Oxidation	Mineralization by means of native catabolic enzymes	Completely eliminates the inhibitors. Uses inhibitor-borne carbon for growth and energy generation	This is by far the most beneficial mechanism. However, it may redirect NAD(P)H and ATP away from product formation early on	^Koopman et al. (2010)^
			Activation of stress response elements	Increased synthesis of fatty-acyl-chain lengths to counter increased membrane permeability and fluidity	Minimizes inhibitor- mediated damage of macromolecules. Maintains membrane integrity. Maintains intracellular pH for optimal metabolic activities	Directs cellular resources to relieve stresses caused by inhibitors, leading to poor growth and products formation	^Yang et al. (2012),^ ^Zheng et al. (2012),^ ^Ujor et al. (2015),^ ^Chang et al. (2021)^
				Increased expression of heat shock proteins, DNA repair enzymes, efflux pumps and membrane transporters			
Aliphatic acids	Acetic acid	C_2_H_4_O_2_; 60.05; −0.28	Activation of stress response elements	Same as described for furanic aldehydes	Same as described for furanic aldehydes.	Same as described for furanic aldehydes	
	Formic acid	C_2_H_4_O_2_; 60.05; −0.28					
	Levulinic acid	C_5_H_8_O_3_; 116.11; −0.49					
Phenolic acids	*p*-coumaric acid	C_9_H_8_O_3_; 164.05; 1.88	Catalytic reduction	Same as described for furanic aldehydes	Same as described for furanic aldehydes	Same as described for furanic aldehydes	^Larsson et al. (2001),^ ^Koopman et al. (2010)^
	Vanillic acid	C_8_H_8_O_4_; 168.14; 1.33	Catalytic oxidation and mineralization	Heterologous laccase-mediated degradation; degradation *via* native catabolic enzymes	Complete removal from the growth environment. Uses carbons from inhibitors as growth and energy source		
	Syringic acid	C_9_H_10_O_5_; 198.17; 1.13					
	Ferulic acid	C_10_H_10_O_4_; 194.18; 1.64	Activation of stress response elements.	Same as described for furanic aldehydes	Same as described for furanic aldehydes	Same as described for furanic aldehydes	
	Cinnamic acid	C_9_H_8_O_2_; 148.16; 2.41					
Phenolic aldehydes	Cinnamaldehyde	C_9_H_8_O; 132.16; 2.12	Catalytic reduction	Same as described for furanic aldehydes	Same as described for furanic aldehydes	Same as described for furanic aldehydes	^Zaldivar et al. (1999),^ ^Dae et al. (2009),^ ^Richmond et al. (2012),^ ^Zhang et al. (2015),^ ^Okonkwo et al. (2019),^ ^Wang et al. (2020)^
	Syringaldehyde	C_9_H_10_O_4_; 182.17; 0.86	Catalytic oxidation and mineralization	Same as described for furanic aldehydes	Same as described for furanic aldehydes	Same as described for furanic aldehydes	
	Vanillin	C8H8O3; 152.15; 1.19					
	4-HBD	C_8_H_8_O_3_; 122.12; 1.39	Activation of stress response elements	Same as described for furanic aldehydes	Same as described for furanic aldehydes	Same as described for furanic aldehydes	

^a^ M_for_ and M_r_ represent the molecular formula and weight (in gmol^−1^) of each inhibitor. *Log P*_*octanol-water*_ is a measure of hydrophobicity of inhibitors and predicts the distribution/partitioning of LDMICs in plasma membranes. Inhibitors with *Log P*_*octanol-water*_ < 1.9 are chaotropic stressors whereas those with a *log P*_*octanol-water*_ >1.9 are strong hydrophobic stressors and can easily diffuse across plasma membrane (Cray et al., 2015). The *log P*_*octanol-water*_ values 4-HBD; 4-hydroxybenzaldehyde.

Trehalose, a non-reducing disaccharide comprising of two glucose molecules linked together by an α,α-1,1 glycosidic bond is used by yeasts and filamentous fungi to counter heat, cold, osmotic, desiccation, oxidative, and ethanol stresses (Crowe et al., 1984; Fillinger et al., 2001; Mahmud et al., 2010; Tapia et al., 2015). Using a combination of phospholipid-targeted metabolomics (phospholipidomics) and transcriptomics, Yang et al. (2012) demonstrated that *S. cerevisiae* challenged with a mixture of acetic acid, furfural and phenol upregulated fatty acid metabolic genes, with concomitant increases in fatty-acyl-chain lengths of phosphatidylcholine and phosphatidylinositol. Perhaps, the cells were countering inhibitor-mediated increase in plasma membrane permeability and fluidity (Yang et al., 2012; Table 1). Thus, it is not surprising that increased accumulation of trehalose following concomitant overexpression of a trehalose-6-phosphate synthase gene (*tps1*) and an aldehyde reductase gene (*ari1*) alongside inactivation of a neutral trehalase gene (*nth1*) in *S. cerevisiae* led to enhanced growth and ethanol yield upon challenge with 10 mM furfural or 30 mM HMF (Divate et al., 2017). These numbers translate to ∼1.0 g/L furfural and 3.80 g/L HMF. Depending on the LBM, LHs can contain as high as 6.0 g/L furfural and >3.0 g/L HMF. Therefore, it is critical to further delineate the mechanistic basis for enhanced HMF tolerance relative to furfural, for the strain of *S. cerevisiae* expressing the trehalose accumulating machinery. This would likely pave the way for additional engineering efforts to increase tolerance to furfural—the major LDMIC in LHs.

**FIGURE 2:**
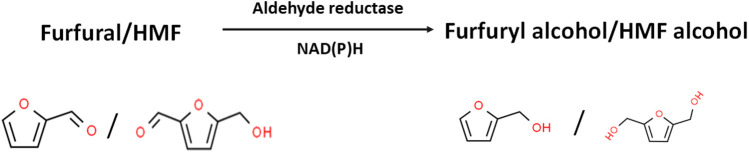
Enzymatic reduction of furfural and HMF to their corresponding alcohols by aldehyde (aldo/keto) reductases. Inhibitor structures were obtained from the ChemSpider database (Available at: http://www.chemspider.com/).

Detoxification of furfural and HMF *via* reduction to their less toxic alcohols is NAD(P)H-dependent (Figure 2; Table 1; Nilsson et al., 2005). Thus, in the presence of high furfural and HMF concentrations, the cells of *S. cerevisiae* (and other organisms) expend considerable amounts of NAD(P)H to reduce both aldehydes to their less toxic alcohols (furfuryl alcohol and HMF alcohol, respectively). Notably, fermentative production of alcohols (ethanol in the case of *S. cerevisiae*) is equally NAD(P)H-dependent. Consequently, detoxification of LDMICs siphons electrons away from target product biosynthesis (Nilsson et al., 2005; Gorsich et al., 2006; Ujor et al., 2014b). However, it is important to highlight that at a low concentration (e.g., 0.25 mg/ml), furfural has been reported to stimulate growth and ethanol yield in *Saccharomyces kluyveri* (Lu et al., 2007). This effect was reversed when the concentration of furfural was increased to 1.00 mg/ml. Similarly, Hovarth et al. (2001) reported increased growth and ethanol production in continuous culture of *S. cerevisiae* challenged with low concentrations of furfural. Notably, in the same study, glycerol yield decreased with increase in furfural concentration, most likely due to increased competition for NADH required for furfural reduction. Ultimately, increasing furfural concentration impairs final alcohol yield. By using reverse genetics (targeted gene deletions), Gorsich et al. (2006) demonstrated that NADPH generation *via* the pentose phosphate pathway (PPP) supplies reductants for furfural detoxification in the form of NADPH. By deleting the PPP genes *zwf1*, *gnd1*, *rpe1*, and *tkl1*, which encode glucose 6-phosphate 1-dehydrogenase (that generates NADPH), 6-phosphogluconate dehydrogenase (that generates NADPH), ribulose-phosphate 3-epimerase, and transketolase, respectively, the resulting strains were significantly impaired for growth in the presence of furfural. Conversely, cloning and overexpressing *zwf1* reversed this phenotype by eliminating sensitivity to furfural. Notably, the *zwf1*-expressing mutants grew at furfural concentrations that previously impaired the growth of *S. cerevisiae* (Gorsich et al., 2006). Despite this observation, which has been confirmed by other authors using different organisms (Allen et al., 2010; Ujor et al., 2014a), it is too simplistic to suggest that increasing NADPH regeneration alone will overcome the toxicities of LDMICs. This is because the toxicities of LDMICs are too complex to be efficiently countered by enhanced NADPH regeneration alone. Notably, LDMICs attack microbial membranes, cause DNA damage and inhibit specific enzymes (Palmqvist et al., 1999; Palmqvist and Hahn-Hägerdal, 2000; Allen et al., 2010). Furthermore, at high furfural and HMF concentrations, which are typical of LHs, both inhibitors are reduced to furfuryl alcohol and HMF alcohol, respectively, both of which exhibit a level of toxicity on fermenting cells, albeit at higher concentrations than their corresponding furanic aldehydes.

**FIGURE 3:**
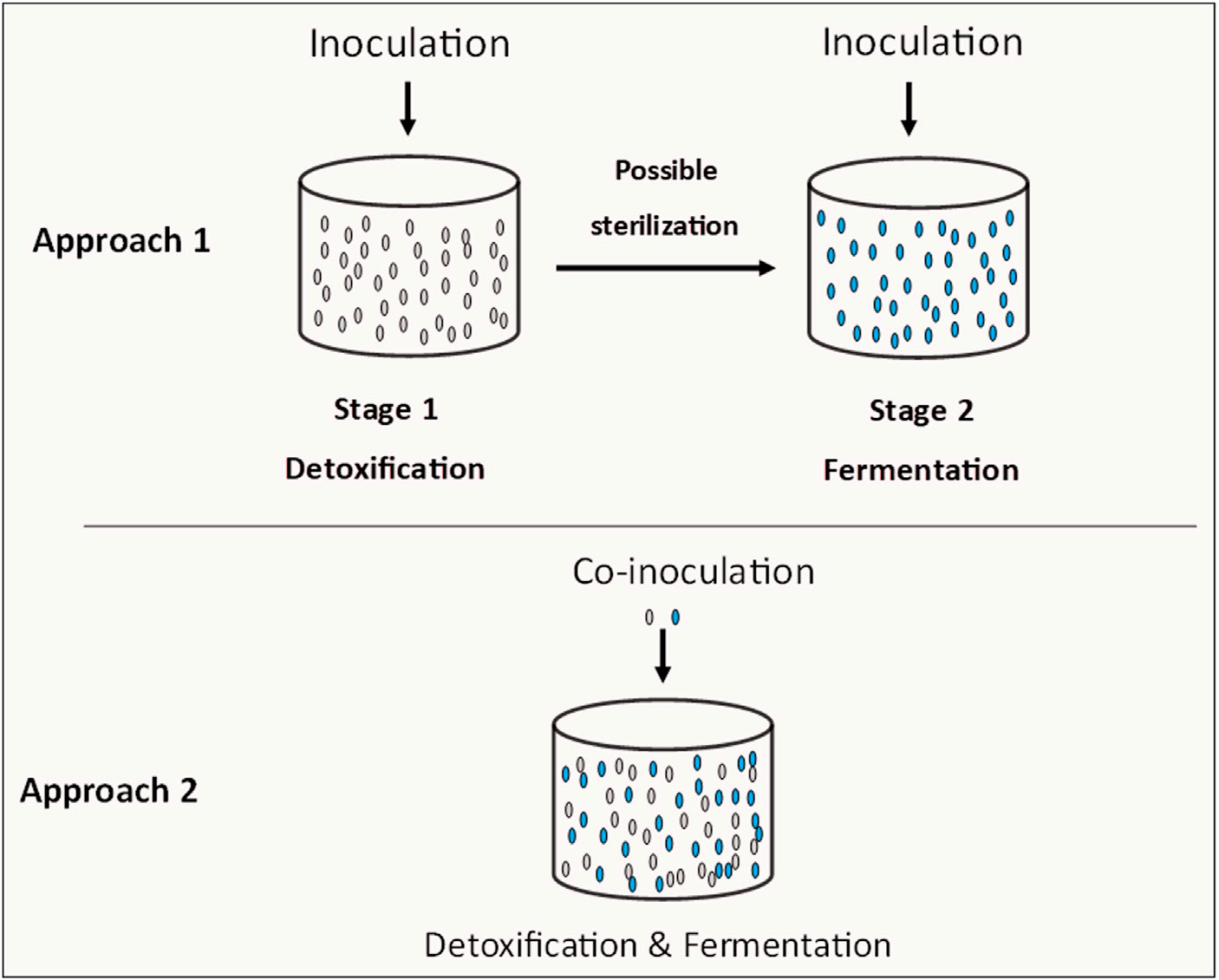
Proposed approach for efficient detoxification of LDMICs and fermentation of LHs to target products by harnessing the power of two or more species or strains.

Zhang and Ezeji (2014) showed that as low as 2 g/L furfuryl alcohol caused >16% reduction in butanol production by *Clostridium beijerinckii*, whilst concentrations greater than 5 g/L inhibited both growth and butanol biosynthesis in this organism. Therefore, it is not implausible to suggest that a similar effect, albeit possibly at higher concentrations of furfuryl alcohol might exist in *S. cerevisiae*. This may account for reduced ethanol concentrations in the cultures of *S. cerevisiae* grown in LHs or furfural-challenged mineral media, despite the reduction of furfural to furfuryl alcohol (Gorsich et al., 2006; Divate et al., 2017). Thus, even a robust strain of *S. cerevisiae* with amplified NAD(P)H-regenerating capacity will still suffer considerable reduction in ethanol production—the ultimate goal of the process. This has significant implications for economical conversion of LBMs-derived sugars to value-added chemicals using *S. cerevisiae* alone. Furthermore, because of their toxicities, LDMICs are first detoxified before target product biosynthesis, thus, high concentrations of LDMICs typically engender a long lag phase, during which product biosynthesis is largely inactive. This represents wasteful use of sugars that are consumed during prolonged detoxification periods, thereby shortening active biosynthesis of the target product. This raises the question as to whether detoxification alone is capable of overcoming the challenge posed by LDMICs to largescale production of cellulosic bio-chemicals. Perhaps, mineralization, wherein, the LDMICs are utilized as carbon sources—either by the fermenting organism, or by a different strain/species—might offer a more robust strategy for economical fermentation of LHs into bio-chemicals. Such an approach though, requires an aerobic stage pre-fermentation, thus, can only be possible with facultative fermentative species. A possible measure to circumvent this is two-stage fermentation approach (Figure 3). This entails extensive engineering of, or isolation of a LDMICs-utilizing strain/species that can be used to first remove the inhibitors prior to fermentation, without consuming LH-borne sugars. Thus, the organism(s) must exhibit a natural penchant for utilizing a wide variety of LDMICs, whilst exhibiting little to no “appetite” for the sugars contained in LHs. Afterwards, the fermentation medium may be sterilized—if a different strain/species is used for LDMICs removal pre-fermentation. More importantly, the detoxification step (*via* utilization of LDMICs) needs to be short (12–18 h) to avoid the attendant cost of a long detoxification stage. Alternatively, the LDMICs-utilizing organism(s) may be co-inoculated with a dedicated fermentative organism, where both can coexist (Figure 3). In this scenario, the fermentative organism will become more active as the concentrations of LDMICs decrease, following utilization by the detoxifying organism/strain. More importantly, concomitant catabolic removal of LDMICs and fermentation of LH-borne sugars to a target product would require the use of an aerobic or facultative detoxifying organism alongside, at least, a facultative species that can tolerate the aerobic stage that is fundamental to microbial utilization of LDMICs.

Strains of *Cupriavidus basilenesis* are prime candidates for two-stage detoxification (*via* inhibitor utilization; Table 1) and fermentation of LHs into target chemicals. Ourselves and others have shown that *C*. *basilenesis* utilizes several LDMICs, without significantly consuming the sugars in LHs (Wierckx et al., 2010; Agu et al., 2016). In fact, *C. beijerinckii* grown in *Miscanthus giganteus* (MG) hydrolysate produced 70% higher concentration of acetone, butanol and ethanol in MG hydrolysate pre-detoxified with *C. basilenesis* ATCC^®^BAA-699, relative to cultures of the same organism grown in non-detoxified MG hydrolysate (Agu et al., 2016). This approach is worth exploring for the production of cellulosic ethanol using *S. cerevisiae*, as well as for production of other chemicals using other organisms. Similarly, recently, we reported that the soil inhabiting nitrogen fixing bacterium *Paenibacillus polymyxa* DSM 365, which produces the important industrial bulk chemical 2,3-butanediol (Kumar and Ujor, 2022) utilizes HMF but not furfural as a sole carbon source (Okonkwo et al., 2021). Therefore, *P*. *polymyxa* DSM 365 represents an attractive candidate for engineering a strain that can concomitantly utilize LDMICs as carbon source and at the same time, produces a target chemical. Such an approach would eliminate the need for a separate detoxification stage using a different organism. However, it is important to note that the ability to mineralize HMF by *P. polymyxa* DMS 365 is accompanied by a long lag phase, which would hamper the economics of the resulting product. Thus, metabolic engineering of *P*. *polymyxa* DSM 365 to extend its LDMIC-based substrates should also include fine-tuning the HMF utilization pathway to more rapidly consume HMF and possibly, other LDMICs.

### Escherichia coli

Because of its fast growth rate and availability of molecular tools for genetic modification to produce different target molecules, *E. coli* is an important organism for largescale operations. As a result, *E. coli* has been extensively researched for concomitant detoxification of LDMICs and target product accumulation during fermentation. Several authors have engineered strains of *E. coli* with enhanced inhibitor detoxification *via* gene deletions and integrations, and expression of plasmid-borne target genes. Notably, as with *S. cerevisiae*, genetic modifications that boost the intracellular availability of NAD(P)H and active reduction of inhibitors (by oxidoreductases; Table 1), particularly, furfural to furfuryl alcohol, have been shown to promote the detoxification of LDMICs and subsequently, fermentation product accumulation. Specifically, overexpressing *yqhD* or *fucO*—either *via* chromosomal integration or by means of a plasmid—both of which encode a furfural reductase in *E. coli* led to enhanced furfural reduction and ultimately, improved growth and ethanol production in furfural-supplemented medium (Wang et al., 2011; Wang et al., 2013). However, overexpression of *fucO* was more effective than *yqhD*. This is because, the protein product of *fucO* is NADH-dependent, which is more abundant under fermentative conditions, whereas *yqhD* encodes a NADPH-dependent furfural reductase, and NADPH is less abundant during fermentation. Similarly, deletion of phosphoglucose isomerase gene (*pgi*) increased growth and ethanol production by *E. coli* in cultures challenged with 1 g/L of each of furfural and HMF (Jilani et al., 2020). The protein product of *pgi* catalyzes the isomerization of d-glucose-6-phosphate (G6P) and d-fructose-6-phosphate (F6P) in glycolysis. Thus, deletion of *pgi* routes sugar metabolism through the PPP, which favors NADPH regeneration—an important reductant for furfural and HMF reduction.

**FIGURE 4:**
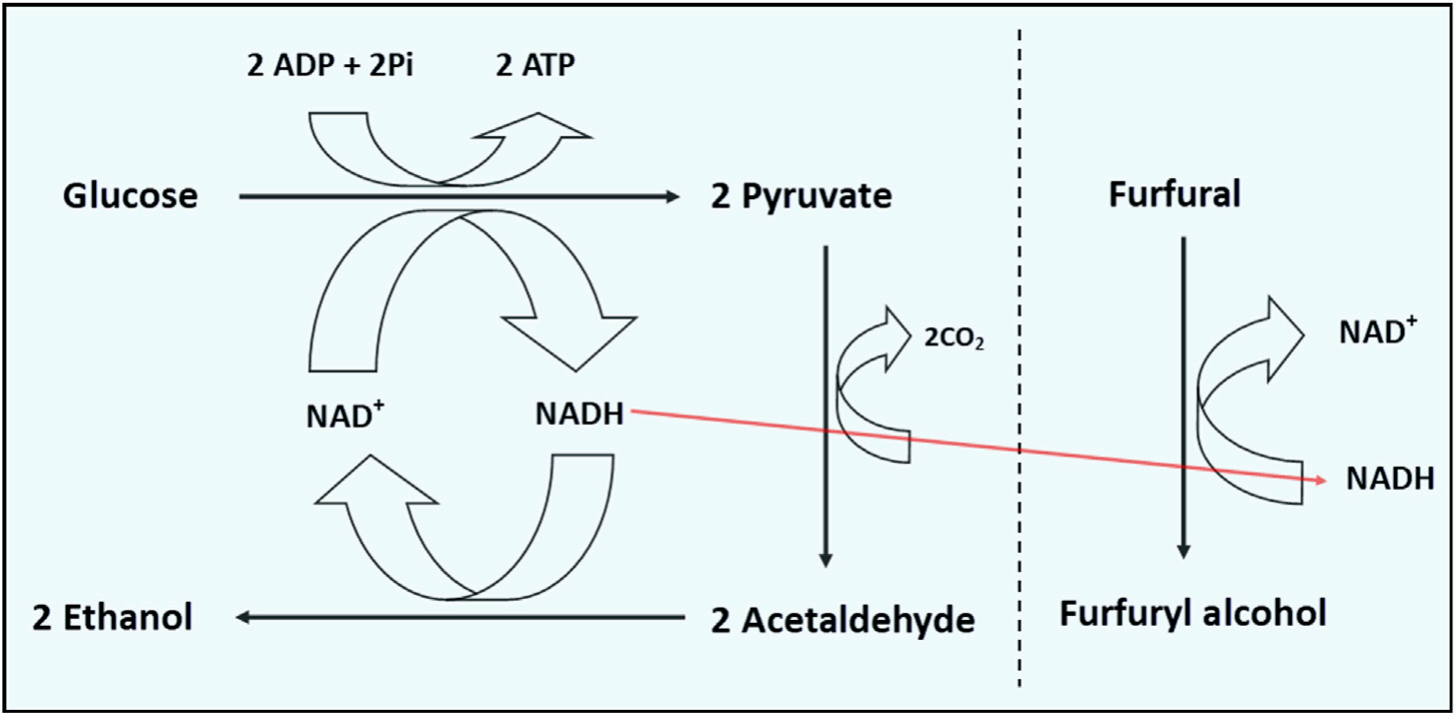
Amplified glycolytic flux supports ATP supply and generation of reductant (NADH) for furfural reduction in *E. coli*.

Using CRISPR-enabled trackable genome engineering (CREATE) to expedite adaptive evolution in *E. coli*, Zheng et al. (2022) evolved a strain that tolerates 4.7 g/L furfural. In this strain, mutations in *rpoB* ^*P153L*^, sRNA *sgrS* and sRNA *arrS* were found to account for the robust tolerance of the resulting strain to furfural. The *rpoB* gene encodes the β subunit of RNA polymerase, which plays an important role in genome-wide transcription (Zheng et al., 2022). Similarly, Small regulatory RNAs (sRNAs) play essential roles in post-transcriptional gene expression regulation and in some cases, controlling large regulons (Zheng et al., 2022). Thus, the authors speculated that mutation of the *rpoB* gene likely accounted for downregulation of the sRNAs *sgrS* and *arrS*. SgrS is a bifunctional sRNA expressed during high intracellular accumulation of glucose-6-phosphate, which functions to blunt glycolytic flux (Wadler and Vanderpool, 2007, 2009). Thus, by downregulating SgrS, NADH-generating glycolysis is less impeded, thereby furnishing the cell with ample reductant (NADH) and energy for furfural reduction (Figure 4; Bobrovskyy et al., 2019; Zheng et al., 2022). The antisense RNA ArrS regulates the expression of the largest transcript of *gadE*, which codes for a transcriptional activator of a glutamate-dependent acid-resistant system in *E. coli* (Aiso et al., 2014; Bianco et al., 2019). Thus, overexpression of ArrS enhances the survival of cells under conditions of high acidity (Aiso et al., 2014). In addition to NAD(P)H depletion, both HMF and furfural have been reported to trigger a sharp drop in ATP levels (Palmqvist et al., 1999; Ask et al., 2013). To counter this, furfural-challenged *C. beijerinckii* elevated acetate and butyrate production, particularly the latter (Ujor et al., 2014b), because production of acetate and butyrate generate ATP (Doremus et al., 1985; Grupe and Gottschalk, 1992). Therefore, it is plausible that furfural might elicit a similar response in *E. coli*, specifically acetate production, in which case improved expression of ArrS mitigates acetate-mediated stresses during growth in a furfural-supplemented culture.

Zheng et al. (2012) demonstrated that plasmid-based overexpression of *thyA*, which codes for thymidylate synthase increased furfural tolerance in *E. coli*. Thymidylate synthase is involved in *de novo* DNA synthesis and furfural causes DNA damage, which explains the basis for *thyA* overexpression-mediated relief of furfural toxicity. The authors also reported a similar effect in *Bacillus subtilis* YB886 and *Zymomonas mobilis* CP4 in which the native *thyA*s were overexpressed. This underscores the multipronged nature of the toxic effects of furfural and indeed, LDMICs which highlights the need for multi-engineering approaches towards constructing microbial ‘juggernauts’ capable of efficient detoxification of, and tolerance to LDMICs. Because the LDMICs in LHs are not instantly removed from the medium during fermentation, equipping fermenting cells or specialized detoxification species/strains—depending on the approach adopted—not only to remove these inhibitors, but also to withstand their effects on cellular macromolecules (such as DNA and proteins) is crucial for economical deployment of LHs in an industrial setting.

Plasmid-based cloning and homologous overexpression of the gene *ycfR*, which encodes a stress responsive element in *E. coli* increased tolerance to acetic acid, furfural and phenol, both separately and together (Chang et al., 2021). The *E. coli* strain expressing an additional copy of YcfR exhibited increased growth, substrate utilization and ethanol production, relative to the wildtype. The protein product of *ycfR* interacts with proteins involved in acid, osmotic, oxidative, and nutrient starvation stress responses, as well as proteins involved in the repair of damaged membrane and DNA (Oughtred et al., 2019). Because furfural causes both membrane and DNA damage (Palmqvist et al., 1999; Palmqvist and Hahn-Hägerdal, 2000; Allen et al., 2010; Yang et al., 2012), it is plausible that overexpression of YcfR elicits rapid and robust recruitment of membrane and DNA repair machineries in response to furfural and phenol (Table 1). Ultimately, this should mitigate toxicity leading to enhanced detoxification of these toxicants. Furthermore, overexpression of YcfR increased tolerance to acetate (Chang et al., 2021). YcfR interacts with proteins involved in acid stress response, which is the likely basis for reduced toxicity of acetate to the strain of *E. coli* expressing an additional copy of *ycfR*.

The above listed studies suggest that *E. coli* deploys a wide assortment of mechanisms to combat LDMICs. Combined cloning—possibly *via* chromosomal integration under the control of appropriate promoters—and expression of the different proteins that have been shown to mitigate inhibitor toxicity in *E. coli* may prove instructive. Because of the multi-pronged toxicities of LDMICs, assembling the different cellular ‘elements’ that dampen inhibitor toxicity in a single strain might allow the strain to simultaneously recover from membrane and DNA damage and enzyme inhibition, while rapidly detoxifying the various LDMICs. Alternatively, engineering a consortium of *E. coli* strains wherein, each strain is genetically optimized to tackle a single or a narrow collection of inhibitors, based on their modes of action equally holds considerable promise.

### Solventogenic *Clostridium* species

Butanol has tremendous promise as a biofuel and as an industrial bulk chemical. Owing to their ability to produce butanol, solventogenic *Clostridium* species have attracted considerable attention over the past 2 decades. Among other factors, lack of a cheap feedstock is major limitation to commercialization of bio-butanol, which makes LBMs attractive for biological butanol production. Most published studies that sought to engineer solventogenic *Clostridium* species for improved fermentation of LHs are based on *C. beijerinckii* NCIMB 8052. Separate integration of the open reading frames Cbei_3974 and Cbei_3907 [encoding an aldo/keto-reductase (AKR) and a short chain dehydrogenase reductase (SDR), respectively] into the chromosome of *C. beijerinckii* NCIMB 8052, under the control of the constitutive thiolase promoter increased tolerance to LDMICs (Okonkwo et al., 2019). However, this increase was more pronounced in the AKR-expressing strain. Notably, the AKR-expressing strain showed greater tolerance to furfural, 4-hydroxybenzaldehyde (4-HBD) and syringaldehyde than the SDR-expressing strain of *C. beijerinckii*. Consequently, the AKR-expressing strain showed higher growth, glucose utilization, and butanol production in media containing 4, 5 and 6 g/L furfural, when compared to the wildtype and the SDR-expressing strain (Okonkwo et al., 2019). Specifically, the AKR-expressing strain of *C. beijerinckii* produced a maximum butanol titer of 12.20 g/L with furfural, whereas the wildtype and the SDR-expressing strains accumulated a maximum titer of 9.20 g/L each. However, when both the AKR-ad SDR-expressing strains were grown in switch grass hydrolysate relative to the wildtype, both engineered strains produced only marginal increases in butanol production. This trend further underscores the need for a radically different approach to the conversion of LBMs to bio-derived chemicals. Similar to *S. cerevisiae* and *E. coli*, a two-stage process will likely benefit LH fermentation to butanol, by broadly eliminating the inhibitors prior to the fermentation stage. This is particularly important in butanol fermentation because the solventogenic clostridia are obligately anaerobic. Anaerobically, furfural and HMF can only be reduced to their respective alcohols, which in turn disrupt butanol biosynthesis (Zhang and Ezeji, 2014).

Ujor et al. (2014a) showed that co-utilization of glycerol with glucose increased furfural tolerance by *C. beijerinckii*—an effect that stems from increased NAD(P)H generation during catabolism of the more reduced glycerol. However, *C. beijerinckii* NCIMB 8052 utilizes glycerol poorly. Therefore, with a view to increasing glycerol utilization by *C. beijerinckii* NCIMB 8052, and hence, increase NAD(P)H generation and in turn, furfural detoxification, Agu et al. (2019) investigated plasmid-based expression of glycerol catabolic genes of *C. pasteurianum* in *C. beijerinckii*. *C. pasteurianum* is an excellent consumer of glycerol as a sole carbon source. Expression of two different glycerol dehydrogenase (*gldA* and *dhaD1*) and a dihydroxyacetone kinase (*dhaK*) genes—either separately or together in different combinations—from *C. pasteurianum* in *C. beijerinckii* NCIMB 8052, resulted in increased glycerol utilization in furfural-supplemented cultures (Agu et al., 2019). Nonetheless, the maximum butanol titers still fell short of commercialization targets. As mentioned earlier, targeting NAD(P)H regeneration or DNA repair alone is not sufficient to effectively combat the multi-pronged deleterious effects of LDMICs. Engineering multiple new functions targeting the different known toxic effects of LDMICs might prove a more successful approach. Additionally, engineering a microbial community in which different strains of the same species or different species target different LDMICs might prove instructive. This is a vastly under-research approach in the effort to convert LBMs to bio-chemicals.

Jiménez-Bonilla et al. (2020) increased the tolerance of *Clostridium saccharoperbutylacetonicum* to furfural (3.5 g/L) and ferulic acid (1.2 g/L) *via* plasmid-based cloning and expression of the gene *srpB*, which codes for a subunit of a *Pseudomonas putida* efflux pump. The mechanistic basis for this may be related to reduced contact with DNA and proteins in *C. saccharoperbutylacetonicum*. Expelling the inhibitors from the cell by means of the *P. putida* efflux pump should minimize their concentrations in the cytoplasm, reduce contact with DNA and proteins—which protects these macromolecules—and most of all, make for rapid detoxification of residual inhibitors in the cytoplasm by native aldehyde reductases. Over the course of fermentation, this is expected to minimize the likelihood of intracellular damages to the cell. Coupling functional expression of such an efflux pump with increased expression of a native aldehyde reductase might further improve the benefits obtained from this strategy. In light of the diversity and concentrations of LDMICs in LHs, such an approach is crucial to efficient conversion of LBMs to bio-chemicals. A study by Suo et al. (2019) highlights the potential of combining different LDMIC-resistant mechanisms in a single strain using metabolic engineering. The authors engineered a strain of *Clostridium tyrobutyricum* that produced >28% greater concentration of butyrate than the wildtype in undetoxified corncob hydrolysate. To achieve this, the authors cloned and co-expressed the earlier described short chain dehydrogenase/reductase (SDR) from *C. beijerinckii* NCIMB 8052, which has been shown mediate inhibitor detoxification in this organism (Zhang and Ezeji, 2013; Zhang et al., 2015; Okonkwo et al., 2019) and a heat chock protein (GroESL) previously reported to enhanced butanol tolerance in *C. acetobutylicum* (Tomas et al., 2003) in *C. tyrobutyricum*. Similar to butanol, GroESL likely dampens the toxicity of butyrate on the cell, which may account for the significant increase in butyrate accumulation following SDR-mediated inhibitor detoxification in the hydrolysate.

### Corynebacterium glutamicum

*Corynebacterium glutamicum* is an important industrial organism, especially for amino acids production. Comparatively, wildtype *C. glutamicum* exhibits greater tolerance to LDMICs than most fermentative bacteria (Sakai et al., 2007; Tsuge et al., 2014; Tsuge et al., 2016; Wen and Bao, 2019; Zhou et al., 2019; Jin et al., 2020). Tsuge et al. (2014) showed that *C. glutamicum* converts furfural to furfuryl alcohol and furoic acid under aerobic condition and to furfuryl alcohol under anaerobic condition, at the expense of NAD(P)H. A study by Tsuge et al. (2016) identified an alcohol dehydrogenase (FudC) as a key player in furfural detoxification by *C. glutamicum*. Notably, deletion of the FudC encoding gene in *C. glutamicum* led to marked reduction in furfural reduction to furfuryl alcohol—with significant reduction in furfural tolerance—by *C. glutamicum* (Tsuge et al., 2016). Similarly, overexpression of an alcohol dehydrogenase encoded by the gene *CGS9114_RS01115* accelerated the rates of furfural, HMF, 4-HBD, vanillin and syringaldehyde reduction by *C. glutamicum* S9114 (Zhou et al., 2019). To increase LHs-conversion to glutamic acid, Wang et al. (2018) deployed adaptive evolution to generate a LDMICs-tolerant strain of *C. glutamicum* S9114. Following 128-day exposure of *C. glutamicum* S9114 to corn stover hydrolysate, the resulting strain exhibited significantly increased detoxification of furfural, HMF, syringaldehyde, 4-HBD, and vanillin (Wang et al., 2018). Furthermore, the LDMICs-tolerant strain utilized higher amounts of glucose resulting in 68% increase in glutamic acid production. The basis for the traits exhibited by this strain were found to be enhanced expression of glycolytic and PPP genes, leading to amplified NAD(P)H regeneration and enhanced overall metabolic flux (Wang et al., 2018).

### Microbial consortia

In an effort to circumvent the inherent challenges of concomitant inhibitor detoxification and fermentation of LH-borne sugars to a target product by one organism, Zou et al. (2021) engineered a strain of *P*. *putida* that exclusively consumes LDMICs, whilst not utilizing the sugars in the hydrolysate. To achieve this, the authors inactivated the glucose import and catabolic machinery of *P. putida* KT2440, which is incapable of xylose utilization. Consequently, the resulting strain was used in a mixed culture to remove the LDMICs in corn stover hydrolysate. Concomitantly, *Bacillus coagulans* was used to ferment the sugars in the hydrolysate to lactic acid. The authors reported 100% removal of furanic aldehydes from the corn stover hydrolysate owing to utilization by the engineered strain of *P. putida* (Zou et al., 2021). Further, 90% of the lignin-derived mono-aromatic inhibitors were detoxified. This led to the production of ∼36 g/L lactic acid, whereas a monoculture of *B. coagulans* without the ‘assistance’ of a synthetic inhibitor-catabolizing *P. putida* failed to ferment corn stover hydrolysate to lactic acid (Zou et al., 2021). However, it is pertinent to highlight that 30% (v/v) corn stover hydrolysate was used for this study. For favorable economics of scale, a significantly higher concentration of the hydrolysate is required. Further, it took 24 h to detoxify the LDMICs in the corn stover hydrolysate. While this is a positive outcome, reducing the duration of the detoxification phase, will further enhance product yield and ultimately improve the economics of the process. Given that both engineered *P. putida* and *B. coagulans* were used in a mixed culture, growth and lactic acid biosynthesis by *B. coagulans* were largely impaired during the first 24 h of the process (during which the inhibitors hampered fermentation by *B. coagulans*). Similarly, Sun et al. (2021) deployed a combination of adaptive evolution and a microbial consortium to achieve 71 g/L lactic acid on corn stover hydrolysate. By enriching and adapting a thermophilic consortium comprising of *Enterococcus*, *Lactobacillus*, *Bacillus*, *Lactococcus*, and *Trichococcus* to harsh environmental conditions, a robust mixed culture was obtained that tolerated 9.74 g/L total LDMICs. Microorganisms exhibit antagonistic interactions in nature. Similarly, they exhibit cooperative interactions where different strains/species work together to achieve a central goal, such that different members of the community perform specific tasks. Because of the complex mixture of LDMICs present in LHs, which exhibit assorted chemicals structures and modes of inhibition, different species/strains are likely to evolve varying capabilities to utilize or merely detoxify different inhibitors, depending on their environmental niches. Therefore, assembling and characterizing a consortium of compatible species and strains may have greater promise than a single strain/species for efficient LH detoxification and fermentation.

## Use of metabolic perturbations to improve the detoxification of LH

### Calcium mitigates the toxicities of LDMICs

Addition of different substances to the fermentation medium can alter the sequences of metabolic activities and cellular responses in different organisms, some of which cause increased tolerance to LDMICs. For instance, using different solventogenic *Clostridium* strains and species (*C. beijerinckii* NCIMB 8052, *Clostridium acetobutylicum* CICC 8016, *C. acetobutylicum* ATCC 824, *Clostridium sporogenes* BE01) and a variety of LBMs (switchgrass, rice straw, corn cob, and corn stover), different authors have shown that addition of CaCO_3_ to the fermentation medium mitigates the toxicities of LDMICs (Liu et al., 2015b; Gottumakkala et al., 2015; Liu et al., 2018; Wu et al., 2019; Su et al., 2020; Olorunshogbon et al., 2022). In all cases, CaCO_3_ increased cell growth and butanol production in the LH. Previously, Han et al. (2013) had demonstrated that Ca^2+^ exerts a pleiotropic effect on *C. beijerinckii*, leading to increased expression of proteins involved in solventogenesis, sugar utilization, nucleic acid and protein stabilization, oxidative and antibiotic stress responses, signal transduction and increased activities of select solventogenic enzymes. Further, different authors have shown that CaCO_3_ supplementation promotes cell growth and target product biosynthesis, using different solventogenic species (Ujor et al., 2014a; Tian et al., 2015; Xie et al., 2015; Luo et al., 2018). Because major LDMICs such as furfural and HMF cause DNA damage and inhibit specific enzymes (Palmqvist et al., 1999; Palmqvist and Hahn-Hägerdal, 2000; Allen et al., 2010), whilst Ca^2+^ increases the expression of proteins that stabilize these macromolecules (Han et al., 2013), it is likely that CaCO_3_ furnishes the cell with ample Ca^2+^ to overcome LDMIC-mediated DNA damage and enzyme inhibition. However, since LDMICs exert other deleterious effects on the cell such as membrane damage (Palmqvist et al., 1999; Palmqvist and Hahn-Hägerdal, 2000; Allen et al., 2010), DNA and protein stabilization alone does not fully account for the effects observed with CaCO_3_ in LHs. It is possible that Ca^2+^ elicits changes in the membrane structure, which mitigates the effects of LDMICs on cellular membranes during growth in the presence of LDMICs. For largescale application purposes however, CaCO_3_ (or CaCl_2_) will likely constitute an economic burden. First, to use them at largescale will attract considerable cost. Second, CaCO_3_ waste is not easy to manage, thus, this approach would diminish the environmental sustainability of such an approach. Therefore, further characterization of the underlying molecular signature of Ca^2+^-supplemented cultures is required, to better identify and delineate cellular elements that may be engineered to achieve the same outcome without the need for Ca^2+^supplementation.

### Culture supplements that activate the nucleic acid salvage pathway dampen LDMIC toxicity

To counter the DNA damaging effect of furfural, Zheng et al. (2012) supplemented cultures of ethanologenic *E. coli*, *B. subtilis*, and *Z. mobilis* with thymine, thymidine and 5,10-methylenetetrahydrofolate, all of which contribute to DNA repair by increasing the availability of deoxythymidine monophosphate (dTMP). All the culture supplements increased furfural tolerance, leading to dose dependent increase in growth and ethanol production in furfural-challenged cultures (Zheng et al., 2012). These results are in agreement with the effect observed with overexpression of *thyA* (thymidylate synthase) gene in the same organisms (Zheng et al., 2012). Apparently, increased ability to repair furfural-induced DNA damage alleviates furfural toxicity (Table 1). Notably, dTMP can also be obtained in the cell *via* the nucleic acid salvage pathway (Zheng et al., 2012). Likewise, addition of allopurinol, a xanthine dehydrogenase inhibitor, xanthine and inosine (a precursor of hypoxanthine) increased furfural detoxification, culminating in increased growth and butanol production in furfural-supplemented cultures of *C. beijerinckii* NCIMB 8052 (Ujor et al., 2015). Further, the authors showed increases in the messenger RNA copies of xanthine and hypoxanthine phosphoribosyltransferases—important purine-salvage enzymes—in allopurinol-supplemented cultures of *C. beijerinckii* challenged with furfural. Additionally, allopurinol dampened the toxic effects of nalidixic acid (a DNA-damaging antibiotic) and improved growth and ethanol production by *S. cerevisiae* cultivated in corn stover hydrolysate (Agu et al., 2018). These findings indicate that blocking the nucleic acid catabolic pathway (i.e., activating the nucleic acid salvage pathway) supplies the DNA repair machinery with the necessary ‘arsenal’ for rapid overhaul of furfural-mediated DNA damage. Consequently, this increases furfural tolerance. Most fermentative and non-fermentative organisms harbor the inherent capacity to detoxify furfural and some other LDMICs, albeit at different rates. Thus, increasing the ability of the cell to alleviate DNA damage buys the native machinery of the cell much needed time to detoxify LDMICs. Notably though, supplementation of allopurinol, xanthine, inosine, thymine, thymidine or 5,10-methylenetetrahydrofolate to the fermentation broth is impracticable for commercial purposes. Therefore, identifying the key genes whose protein products are central to the payoffs obtained with these supplements may help to construct superior strains that can more efficiently and economically convert LHs to target chemicals.

### Cheap glycerol may hold the key to bioconversion of LHs to target products

Contrary to CaCO_3_, allopurinol, thymine, thymidine and 5,10-methylenetetrahydrofolate, xanthine and inosine, which mitigate furfural toxicity without being actively involved in furfural detoxification, glycerol catabolism fuels the activities of furfural detoxifying enzymes. Ujor et al. (2014b) demonstrated that co-utilization of glycerol and glucose expedited furfural reduction to furfuryl alcohol by *C. beijerinckii*. Furfural and other LB-derived furanic and phenolic aldehydes are reduced to the corresponding alcohol by aldehyde reductases. In fact, some organisms harbor multiple copies of these reductases. Consequently, inability to tolerate LDMICs is often not for lack of enzymes required for inhibitor detoxification. On the contrary, insufficient supply of NAD(P)H is often the impediment, as these reductases are NAD(P)H-dependent. Glycerol catabolism generates two extra moles of NADH, when compared to consumption of equal amount of glucose on a molar basis (Neijssel et al., 1975; Lin 1976). Thus, supplementing glycerol in cultures of *C. beijerinckii* challenged with 4–6 g/L furfural, led to increased availability of NAD(P)H, improved growth and enhanced butanol production (Ujor et al., 2014a). Further, a 1:1 ratio mixture of glycerol and spruce biomass hydrolysate (SBH) led to significant increases (up to 2.6-fold) in the production of 1,3-propanediol and butanol by *C. pasteurianum* DSMZ 525 relative to the cultures of the same organism grown in SBH without glycerol supplementation (Sabra et al., 2014). It is important to note though that only a narrow range of fermentative organisms efficiently utilize glycerol, which is currently available in large amount as a waste product of the biodiesel industry. In fact, in the two studies that deployed glycerol to increase the detoxification of LDMICs, glycerol utilization stalled or stopped after glucose was depleted (Ujor et al., 2014b; Sabra et al., 2014). Therefore, expanding the range of fermentative organisms that can efficiently utilize this cheaper feedstock to mitigate the toxicity of LHs by metabolic engineering or screening for novel glycerol utilizing strains/species will likely help to expedite largescale utilization of LBMs at commercial scale.

## Targeting lignin-derived aromatic inhibitors for detoxification and as substrates to produce value-added chemicals

As mentioned earlier, nimble coupling of inhibitor-borne carbons to product biosynthesis represents an attractive strategy for valorization of LBMs. Notably, lignin accounts for 15%–40% of LBMs (Ragauskas et al., 2014; Chen and Wan, 2017; Xu et al., 2019). In fact, production of 1 L of cellulosic ethanol is accompanied by the generation of ∼1 kg of lignin as byproduct (Xu et al., 2019). Furthermore, globally, the pulp and paper industry generates approximately 50 million tons of lignin per year (Xu et al., 2019). This represents a vast repository of untapped renewable carbon that could be harnessed to produce value-added chemicals. Additionally, during pretreatment of LBMs, lignin-derived aromatic compounds such as 4-HBD, vanillin, vanillic acid, *p*-coumaric acid, syringaldehyde and ferulic acid, among others are co-generated with sugars and furanic aldehydes (Richmond et al., 2012; Wang et al., 2020). Although they occur at lower concentrations than furanic aldehydes in LHs, lignin-derived aromatic inhibitors exert severe toxicities on fermenting organisms, often impeding target product biosynthesis. The severity of these effects vary amongst different organisms. For instance, Zaldivar et al. (1999) reported significantly higher toxic effects of the aromatic aldehydes 4-HBD, syringaldehyde, and vanillin on the growth of *E. coli*, relative to furfural and HMF (furanic aldehydes) on a weight basis. Similarly, Dae et al. (2009) demonstrated that 1 g/L *p*-coumaric acid, ferulic acid, syringaldehyde, and vanillin considerably inhibited the growth of *Clostridium beijerinckii* NCIMB 8052 accompanied by little or no butanol production. Conversely, Richmond et al. (2012) showed that 0.2–1.0 g/L syringaldehyde marginally increased the growth of *C. beijerinckii* NCIMB 8052 when supplemented in the exponential (acidogenic) phase. However, when supplemented in the early solventogenic phase, syringaldehyde elicited severe inhibition of growth, glucose uptake and utilization and acid re-assimilation—which is crucial for butanol biosynthesis (Richmond et al., 2012). Consequently, ABE fermentation was prematurely terminated.

The toxicity of lignin-derived aromatic inhibitors is ascribed in part to their interactions with hydrophobic sites on microbial membranes, which increases membrane fluidity, thereby leading to leakage of cytoplasmic constituents (Giovani et al., 2006). Therefore, rational engineering of microbial membranes to evade these interactions—especially when combined with engineering approaches targeted at direct detoxification of aromatic inhibitors—is a poorly researched area in the efforts to valorize LBMs. For example, when compared to the wildtype, a strain of *C. beijerinckii* NCIMB 8052 expressing an additional copy of chromosomally integrated AKR exhibited improved tolerance to 4-HBD and syringaldehyde (Okonkwo et al., 2019). With the supplementation of 1 g/L of both aromatic inhibitors, the engineered strain produced more butanol that the wildtype. However, the maximum butanol concentrations were 2.5 and 4.0 g/L with syringaldehyde and 4-HBD, respectively (Okonkwo et al., 2019). To increase butanol yield to levels that would warrant commercialization, it is critical to significantly extend these efforts, especially given the fact that these inhibitors which occur together in LHs were tested separately in the referenced study. Thus, combining engineering of microbial membranes to minimize the toxicity of aromatic inhibitors with upgrading the catalytic machineries for aromatic inhibitor detoxification or metabolism would likely minimize inhibitor-mediated stresses, whilst accelerating inhibitor removal from the fermentation medium. Towards this goal, bacteria that inherently metabolize lignin-derived aromatics will play a crucial role.

Different species of *Rhodococcus* have been used to produce economically important lipids using a variety of lignin-based feedstocks such as kraft lignin, alkali-extracted lignin, lignin from combinatorial pretreatment and a mixture of 4-hydroxybenzoic acid, vanillic acid and glucose as co-substrates (Kosa and Ragauskas, 2013; Wei et al., 2015; Zhao et al., 2016; He et al., 2017a; He et al., 2017b; Goswani et al., 2017; Shields-Menard et al., 2017; Xu et al., 2019). Furthermore, bacterial conversion of lignin-derived aromatics to polyhydroxyalkanoates—biopolyesters accumulated as energy reserve by a variety of microbes—which are currently attracting attention as renewable and biodegradable alternative to conventional plastics has been demonstrated (Linger et al., 2014; Kumar et al., 2017; Si et al., 2018; Xu et al., 2019). It is likely that engineered or native bacteria that metabolize lignin-derived aromatics (such as *Rhodococcus* sp., *Cupriavidus basilensis* B-8, *Pandoraea* sp., and *P. putida* KT2440-CJ124) and those that synthesize these aromatics (such as *Shewanella putrefaciens*, *E. coli* and *Burkholderia glumae* BGR1; Johnson and Beckham, 2015; Jung et al., 2016; Wu et al., 2017; Sharma et al., 2018; Xu et al., 2019) harbor robust mechanism to tolerate lignin-derived aromatics, in addition to superior aromatic catabolic machineries. Mining the genomes of these organisms will likely unearth the underlying genetic and enzymatic repertoires, the functions of which can be amplified or tweaked in native organisms to accelerate the catabolism of lignin-derived aromatics, as well as increase tolerance to these compounds. More importantly, these functions can be engineered into non-native organisms that produce other important value-added chemicals. Such an approach promises to mitigate the toxicities contributed by lignin-derived aromatic compounds in LHs, as well as pave the way to funnel carbons from lignin-derived aromatic compounds towards biosynthesis of assorted target chemicals.

## Improving tolerance to LDMICs by adaptive evolution

While metabolic engineering allows researchers to make specific changes in the molecular repertoire of microorganisms geared towards increasing tolerance to LDMICs, microorganisms ‘rearrange’ their genetic and molecular armory against LDMICs during adaptive evolution to better evade and/or combat the stresses posed by these inhibitors. In addition to adaptive evolution of *C. glutamicum* to LDMICs discussed earlier, several organisms have been adapted to tolerate higher than normal concentrations of an assortment of LDMICs. For example, Kurosawa et al. (2015) generated a strain of the triacylglycerols (potential precursors of lipid-based fuel)-producing *Rhodococcus opacus* that is tolerant to lignin, 4-HB and syringaldehyde. To obtain this strain, the authors subjected electro-competent cells of *R. opacus* to electroporation, followed by cultivation on agar medium containing to lignin, 4-HB or syringaldehyde for 14 days. Strains that adapted to the inhibitors exhibited robust growth after the adaptation period and were serially transferred to increasing concentrations of the inhibitors in liquid medium. Relative to the parent strain, the evolved strain exhibited two- to 3-fold greater resistance to lignin, 4-HB, and syringaldehyde, with markedly shorter lag phases in inhibitor-supplemented media. Similarly, Wang et al. (2021) evolved a strain of *Yarrowia lipolytica* that tolerates 1.5 g/L ferulic acid, whereas 0.5 g/L of the same inhibitor proved lethal to the control strain. Transcriptomic analysis of the ferulic acid-adapted strain of *Y. lipolytica* revealed four genes (*YALI0_E25201g*, *YALI0_F05984g*, *YALI0_B18854g*, and *YALI0_F16731g*) that were markedly upregulated in response to ferulic acid. Cloning and overexpression of these genes in separate strains of *Y. lipolytica* improved tolerance to ferulic acid. Additionally, the recombinant strains of *Y. lipolytica* expressing *YALI0_E25201g*, *YALI0_B18854g*, and *YALI0_F16731g* also showed considerable increases in tolerance to vanillic acid. In a different study, growing *S. cerevisiae* over 300 generations in mineral medium supplemented with increasing concentrations of furfural led to strains with significantly reduced lag phases in furfural-containing media and in hydrolysate-supplemented medium (Heer and Sauer, 2008). The furfural-adapted strains of *S. cerevisiae* grew at concentrations of LH that effectively killed the parent strain.

Due to some similarities in the modes of action of furanic and phenolic LDMICs, microorganisms appear to develop cross resistance to different inhibitory compounds during adaptive evolution with a single or a few inhibitors. Notably, LDMICs are mostly catalytically detoxified to their less toxic alcohols in an NAD(P)H-dependent manner. Hence, both furanic and phenolic aldehydes and acids tend place a similar burden on the redox state of the cell. However, there are some differences in the modes of action of different LDMICs. For example, more hydrophobic inhibitors such as furfural, cinnamaldehyde and ferulic acid are more likely to strongly interact with hydrophobic sites on microbial membranes than HMF and 4-HBD, which are relatively more hydrophilic. Apparently, such variations in interaction patterns with microbial macromolecules bring about salient differences in the potencies of different LDMICs. Therefore, adaptive evolution approaches that use different LDMICs that are separately supplemented to the culture medium may not confer molecular adaptations able to sufficiently counter the numerous LDMICs present in LHs. Thus, to generate a strain that broadly tolerates the glut of inhibitors in LH, Liu et al. (2020) challenged *C. beijerinckii* NCIMB 8052 with increasing concentrations of corn stover hydrolysate (CSH). The resulting strain (CIBTS1274A) exhibited superior growth and solvent production profiles in non-detoxified CSH, when compared to the parent strain. To produce sugar acids from LH, *Gluconobacter oxydans* was evolved to tolerate CSH (Jin et al., 2019). The CSH-tolerant *G. oxydans* was obtained by alternating the growth of this organism between inhibitor containing CSH and detoxified CSH for 420 days. The LH-tolerant strain showed several folds faster conversion of CSH-borne sugars to sugar acids, achieving complete conversion of sugars in CSH to their corresponding acids (Jin et al., 2019). Similarly, following a 198-day cultivation of *Zymomonas mobilis* in CSH, a strain tolerant to the LDMICs in CSH evolved (Yan et al., 2021). The resulting strain demonstrated considerably higher capability to detoxify phenolic inhibitors in CSH leading to ∼22% increase in ethanol yield when grown in CSH. Transcriptional analysis revealed that the evolved strain showed greater expression of the gene *ZMO3_RS07160* encoding an SDR family oxidoreductase. SDRs have been strongly implicated in the detoxification of LDMICs (Zhang and Ezeji, 2013; Zhang et al., 2015; Okonkwo et al., 2019).

By sequentially combining interspecies hybridization and adaptive evolution using ammonia fiber explosion pretreated CSH, Peris et al. (2017) generated strains of *Saccharomyces* species with enhanced capabilities for cellulosic ethanol production. Similarly, exposure of *S. cerevisiae* to increasing concentrations of dilute acid pretreated CSH over 463 generations engendered genetic mutations that conferred enhanced tolerance to CSH-borne LDMICs (Almario et al., 2013). Interestingly, whereas the resulting strains exhibited enhanced growth and ethanol production in CSH, they poorly tolerated individual inhibitors supplied separately in a mineral medium. This underscores the complex nature of the interactions between fermenting cells and LDMICs in different LHs. More importantly, it sheds light on the need to incorporate LHs in adaptive evolution efforts geared towards efficient conversion of biomass-derived sugars to fuels and chemicals. Even where individual inhibitors are used at the start of adaptive evolution, it is important to significantly incorporate LHs in the adaptation scheme downstream. Further, a one-size-fits-all approach may not provide the much needed answers to biological conversion of LHs to value-added chemicals. Hence, as opposed to engineering or evolving a strain that can ferment all LHs, it might be more instructive to focus on individual strains/species that are supremely adapted to metabolizing specific LHs from particular plant sources. Apparently, each plant species harbors a unique chemistry that influences the composition of its hydrolysate and as such, the effects on different microbial species and strains. Therefore, a microbe that thrives on poplar hydrolysate may grow extremely poorly in red cedar hydrolysate.

## Conclusion and future perspectives

To realize the potential of LBMs as feedstocks in biomanufacturing, adroit and radical strategies are required to overcome current challenges. First, contrary to reliance on single strains/species, scarcely studied microbial consortia deserve considerable attention. Given the diversity of LDMICs present in LHs, multiple microbial players might offer the greater metabolic dexterity needed for enhanced detoxification of LDMICs than pure cultures. Second, combining microbial consortia with metabolic engineering holds significant promise. For instance, careful engineering of an overproducer of a particular product, which can then be deployed in conjunction with a consortium adept at inhibitor detoxification might eliminate the need to engineer a single strain that both efficiently detoxifies and overproduces a target product. Given the complexity of microbial metabolic networks, attempts to engineer an overproducer might disrupt efforts geared towards engineering robust detoxification traits in the same strain. Third, some medium supplements have shown considerable promise to elicit improved tolerance to LDMICs. However, the molecular bases for these responses have not been fully defined. Thus, there is need to further characterize the molecular repertoires of Ca^2+^, allopurinol, thymine and thymidine—among others—supplemented cultures. The findings of such studies will arm metabolic engineers with new targets to explore, in the effort to obtain robust strains for purposes of both inhibitor detoxification and bio-chemical overproduction. Additionally, combining metabolic engineering with adaptive evolution promises to simultaneously harness an ever growing wealth of tools to fine-tune the molecular armory of microorganisms and the vast ability of microbes to retool their biochemical arsenal in harsh environmental conditions such as LHs, to develop superior biocatalysts for efficient conversion of LBMs to value added chemicals.
